# 3D‐printed patient‐specific instrumentation and the freehand technique in high‐tibial osteotomy: A prospective cohort‐comparative study in an outpatient setting

**DOI:** 10.1002/jeo2.70088

**Published:** 2025-01-20

**Authors:** Giovanni Grillo, Alexandre Coelho, Xavier Pelfort, Ferran Fillat‐Gomà, Arnau Verdaguer Figuerola, Sergi Gil‐Gonzalez, Juan Manuel Peñalver, Christian Yela‐Verdú

**Affiliations:** ^1^ Department of Orthopedics and Traumatology, Knee Unit, Parc Taulí Hospital Universitari, Institut d'Investigació I Innovació Parc Taulí (I3PT‐CERCA) Universitat Autònoma Barcelona (UAB) Sabadell Spain; ^2^ Fondazione Policlinico A. Gemelli Orthopedic Department Roma Italy; ^3^ 3D Surgical Planning Lab, Parc Taulí Hospital Universitari, Institut d'Investigació I Innovació Parc Taulí (I3PT‐CERCA) Universitat Autònoma de Barcelona (UAB) Sabadell Spain

**Keywords:** 3D printing, knee, open wedge knee osteotomy, patient‐specific instrumentation, tunnels

## Abstract

**Purpose:**

Tibial valgus osteotomy has shown to be a successful and cost‐effective procedure. The advent of image processing and three‐dimensional (3D) printing is an interesting tool for achieving more accurate and reproducible results. The aim of our study was to compare the accuracy of the conventional technique and the use of customized guides in the correction of tibial deformities in tibial varus patients, the surgical and clinical benefits, and the impact of treatment in the outpatient setting.

**Methods:**

A prospective cohort of 30 patients who underwent tibial valgus osteotomy were selected and randomized into two groups (3D‐printed guidewires and conventional techniques). All patients underwent a complete radiological study to plan the surgical procedure. During the surgical procedure, the surgical time and X‐ray exposure were analysed. The following results were evaluated: surgical time and X‐ray exposure, the correlation between the planned correction and the correction obtained at 3 post‐operative months, pre‐ and post‐operative knee injury and osteoarthritis outcome score (KOOS) value at 3 and 12 months, and differences between the two groups in terms of the correction obtained.

**Results:**

Radiation exposure in the ‘3D‐guide’ group was significantly different (8 [±4.51], *p* < 0.05), whereas surgical time was not significantly different between the control and guide 3D groups (60.69 [±8.89] and 53.43 [±11.69], respectively). At the 3‐month follow‐up, the post‐operative hip–knee–ankle and post‐operative mechanical–proximal–tibial angle were not significantly different (*p* > 0.05). At 3‐ and 12‐month post‐surgery, the Knee Injury and Osteoarthritis Outcome Score (KOOS) did not significantly differ between the conventional technique and the 3D‐guide technique (*p* > 0.05). The KOOS at 3 months were 87.86 (±5.64) for the control group and 88.46 (±3.53) for the 3D‐guide group, while at 12 months they were 91.5 (±5.72) for the control group and 88.57 (±8.81) for the 3D‐guide group.

**Conclusion:**

Customized 3D‐printed guides do not permit better correction or functional results than the conventional technique; rather, they reduce surgical time and intraoperative radiation exposure.

**Level of Evidence:**

II.

**Abbreviations**
3D: three‐dimensional
3D‐PSI: 3D Guides Printed
BMI: body mass index
CT scan: computed tomography scan
FHT: free‐hand technique
HKA: hip–knee–angle
HTO: high tibial osteotomy
JLCA: joint line convergence angle
KOOS: Knee Injury and Osteoarthritis Outcome Score
MPTA: mechanical proximal tibial angle
OWHTO: open wedge high tibial osteotomy
SD: standard deviation

## INTRODUCTION

Open wedge high tibial osteotomy (OWHTO) has been described as an efficient surgical treatment that can provide pain relief and decelerate the progression of medial osteoarthritis (OA), preserving the bone stock for patients with moderate medial knee OA and lower leg malalignment [5, 15].

The literature has shown good results for high tibial osteotomy (HTO) procedures to correct malalignment and restore patient function. In recent years, the use of OWHTO (opening‐wedge HTO) has increased in popularity among surgeons because of its greater reproducibility, accuracy and modern planning techniques. OWHTO is also an excellent option for delaying knee arthroplasty and, at a later stage, does not prevent total knee arthroplasty surgery [14, 15, 18].

It is also challenging to achieve satisfactory angular correction, particularly with regard to the mechanical proximal tibial angle (MPTA) and hip‒knee–angle (HKA). Notably, treatment is most effective when optimal angular correction is attained. However, achieving the desired preoperative correction is often challenging with the conventional freehand technique (FHT). Additionally, traditional instrumentation may be limited by the patient's phenotype, especially when multiplanar correction is needed. As a result, careful patient selection and precise surgical techniques are crucial factors in achieving successful outcomes [7, 21, 33].

Radiology studies have revealed that over‐ or under‐correction is common and that opening‐wedge osteotomies are more significant than closing‐wedge osteotomies [18]. Computer‐assisted surgery and patient‐specific cutting guides following three‐dimensional (3D) planning could help surgeons avoid miscorrections [23, 25] while increasing the rate of intraoperative and post‐operative specific or nonspecific complications. Several studies have reported that the complication rate following medial OWHTO ranges from 1.9% to 55% [32]. Chaouche et al. [6] reported that the overall complication rate was 32% up to 2 years post‐surgery, with most cases being classified as minor events (28%).

Recent improvements in computed tomography (CT) deformity analysis and 3D printing tools have enabled the advancement of PSI to achieve optimal correction safely and reliably [13, 22, 25]. The purpose of this study was to compare the accuracy of using a 3D‐printed model‐guided plate with that of traditional techniques for performing OWHTO. The secondary purpose was to evaluate the short‐term clinical outcomes, total surgery time in minutes and fluoroscopy time in seconds for patients undergoing OWHTO, as demonstrated in studies in the modern scientific literature [22].

By comparing all those variables, this study aimed to provide valuable insights into the potential benefits of utilizing a 3D‐printed model‐guided plate for OWHTO in an outpatient setting.

## METHODS

The study received institutional review board approval from the ethics committee (OTV‐3D/CIR2019007). Written consent was obtained from all participants prior to the commencement of the research.

A single‐centre, controlled, randomized, prospective clinical trial was conducted with a sample of 30 patients affected by constitutional genu varum. The aim of this study was to compare the clinical and radiographic outcomes of patients who underwent OWHTO via 3D‐printed, patient‐specific implant guides (3D‐PSI) with those of patients who underwent the conventional technique. The study was conducted in a third‐level centre, with two senior surgeons performing the OWHTO procedures [3].

The study involved 30 patients with constitutional genu varum and medial compartment involvement (Ahlbäck ≤2) requiring medial OWHTO. The study was open‐label for investigators but blinded for patients. These patients were randomized and assigned to undergo surgery either with a customized cutting guide designed individually on the basis of a prior CT scan or via the conventional technique (control group).

The technique was chosen before the intervention through random assignment to one of the groups, taking into account the time required to produce the 3D guide. The randomization process was designed to minimize bias. A randomization list was created, with each assignment number corresponding to a specific type of treatment (either the customized guide or the conventional technique). The treatment type assigned to each number was recorded in a document, which was placed in sealed opaque envelopes kept in the orthopaedic and trauma departments. The envelopes were assigned to patients in consecutive order.

The expected sample size was calculated by accepting an alpha level of 0.05 and a beta level of less than 0.2 in a bilateral contrast. Eighteen subjects were selected for each group to detect a difference of 3 units or more, assuming a common standard deviation of 3. A loss‐to‐follow‐up rate of 10% was estimated; however, owing to the COVID‐19 pandemic, six patients were ultimately lost during the study.

A total of 30 patients, with a mean age of 50.80 (±8.67) years (Table 1), were enroled between November 2019 and December 2022, following strict inclusion and exclusion criteria [2, 11] (Table 2).

**Table 1:** Patient characteristics.

	Conventional technique group, *N* = 15	3‐D guide group, *N* = 15	Total, *N* = 30
Age in years	49.19 (10.17)	52.64 (6.45)	50.8 (8.67) (*p* 0.271)
BMI	29.93 (5.74)	29.87 (4.25)	29.91 (5.01) (*p* 0.91)
Ahlbäck Classification			
I	5 (33.3%)	7 (46.7%)	12 (40%)
II	10 (66.7%)	8 (53.3%)	18 (60%)

*Note*: Median (IQR).

Abbreviations: BMI, body mass index; IQR, interquartile range.

**Table 2:** Inclusion and exclusion criteria.

Inclusion criteria	
Varus tibial deformity	160° < HKA > 175°
Symptoms	Pain
Osteoarthritis Ahlback	I–II
ASA score	I–II
Signature informed consent to surgery	Yes

**Exclusion criteria**	
Clinical history	Previous knee fractures, previous knee surgery, articular deformities and vascular pathology
Previous knee implants	For example, plates and screws
Signature informed consent to surgery	No

Abbreviations: ASA, American Society of Anesthesiologists; HKA, hip–knee–angle.

All patients underwent comprehensive clinical and imaging evaluations. The imaging assessment included knee X‐rays with anteroposterior, lateral and Rosenberg and Schuss views [30], as well as full‐weight‐bearing long‐leg standing anteroposterior radiographs and CT scans. Miniaci's method [24] was used to plan the correction angle, using Mikulicz's weight‐bearing line projected through the anterolateral tibial spine of the knee.

The imaging evaluations were utilized for surgical planning and assessment of the correction achieved. All patients received an arthroscopic evaluation of the knee prior to osteotomy to assess the articular surface condition and concomitant injuries.

After a follow‐up period of 3 months, imaging evaluation was performed to quantify the correction obtained.

The degrees of correction achieved in both groups by measuring the HKA, the lateral distal femoral angle, the joint line convergence angle (JLCA) and the MPTA were recorded. The pre‐surgery and post‐surgery results, as well as the difference between the planned and obtained values, were compared. Table 3 shows the HKA measurements before the intervention, the planned degrees of correction and the HKA values after the intervention.

**Table 3:** Measures of the HKA before the operation, the planned degrees of correction and the post‐intervention HKA degrees.

	HKA	Planned degrees of correction	HKA post
1	169	13.4	176.7
2	173	9.8	183
3	169	12.5	179
4	174	7.9	178
5	174	8.8	179.76
6	172	11.7	179.35
7	170	12.9	179.1
8	171	10.2	177
9	171	9.1	177.1
10	170	11.2	179.23
11	167	14.4	177
12	176	7.2	179
13	169	12.4	179.39
14	172.7	8.7	178.4
15	161.6	14	178
16	169	11.2	177
17	173	8.1	177.65
18	174	9	178
19	175	7	182
20	174	10	177.52
21	170	13	179.77
22	172	10.68	178.35
23	168	13	178.71
24	170	11	179.7
25	172	11.94	176.67
26	174	9.74	176.68
27	176	4.74	177.2
28	173	10	177.87
29	171	9.46	178
30	169	15	179.39

Abbreviation: HKA, hip–knee–angle.

To assess clinical outcomes before surgery, at the 3‐month and 1‐year follow‐up periods, the Knee Injury and Osteoarthritis Outcome Score (KOOS) [29] was used in both groups. Data on surgical time in minutes and radiation exposure during the procedures were collected. These parameters were measured to compare the efficiency and safety of the two techniques.

In the 3D‐Guide group, surgical planning and the utilization of 3D‐PSI were based on the preoperative CT scan with the following characteristics: a minimum slice thickness of 0.625 mm (1 mm max), contiguous or overlapping slices (no gaps allowed), a matrix size of 512 × 512, a voxel size of 0.6, and an anatomical region default kernel (standard or high resolution) of 90–120 kVp. We postprocessed the images into a mesh‐volume file format and conducted targeted segmentation of the region of interest via Materialize Mimics Medical 21.0 (part of the Mimics Innovation Suite, Materialize NV). The resulting mesh files were then exported to 3‐matic Medical 13.0 (also part of the Mimics Innovation Suite, Materialize NV) for the purpose of in‐house surgical planning and the design of surgical guides. This process was overseen by the biomedical engineering team at the 3D Surgical Planning Lab, which operates under the legally obtained custom manufacturing licence FMP004CAT.

**Figure 1:**
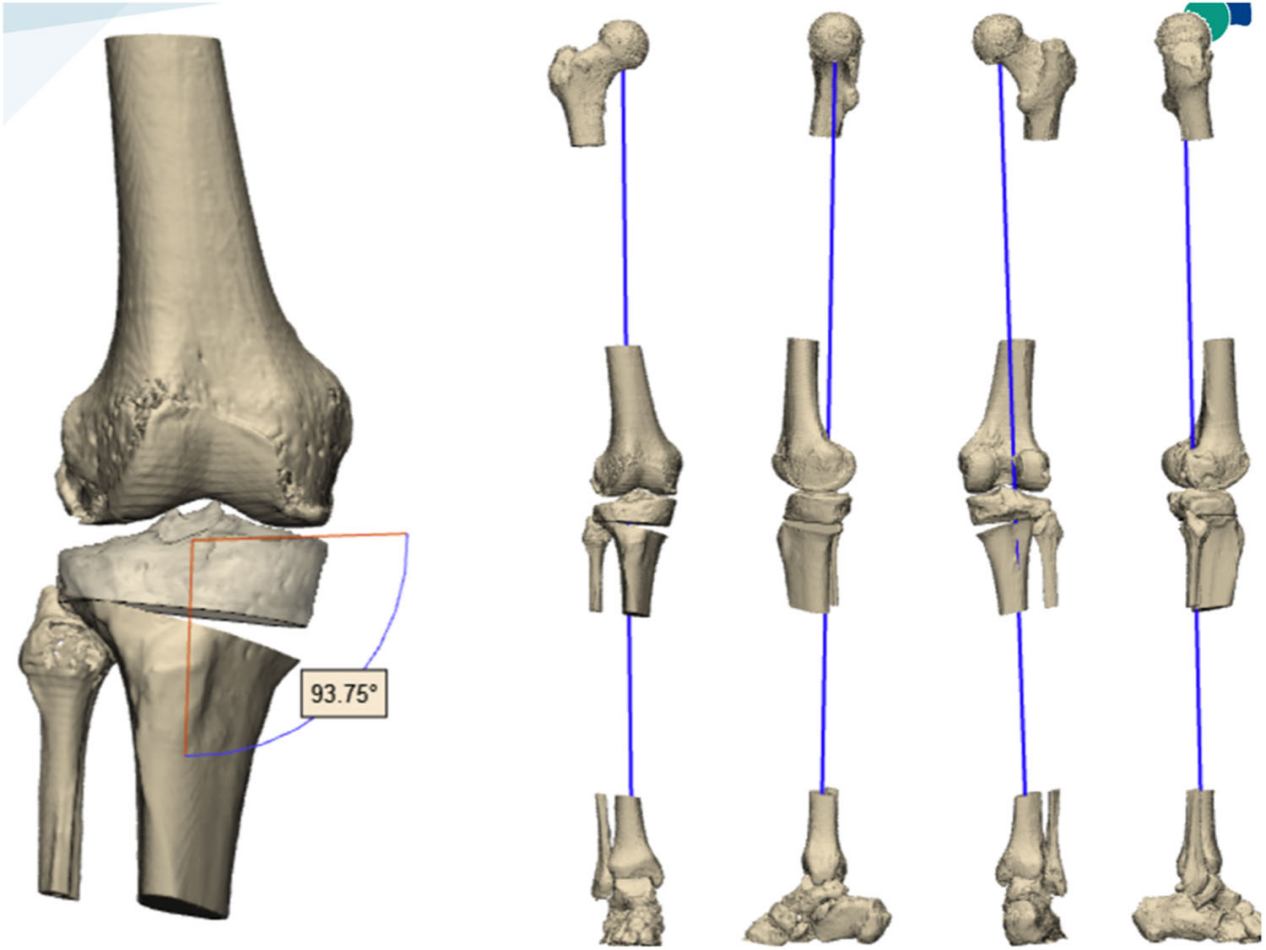
Results on the MPTA and results on the mechanical axis. MPTA, mechanical proximal tibial angle.

Surgical 3D planes were created via the design software 3‐matic Medical 13.0, following the biomechanical studies analysing the stability of OWHTO by Cofaru et al. [8] and Fortier et al. [16] (Figure 1).

Following the biomechanical studies analysing the stability of OWHTO by Cofaru et al. [8] and Fortier et al. [16] and using the Miniaci technique [24], the Fujisawa point or the lateral tibial spine was used as a pin for surgical planning in both groups (all surgical plans were performed via the design software 3‐matic Medical 13.0) (Figure 1).

**Figure 2:**
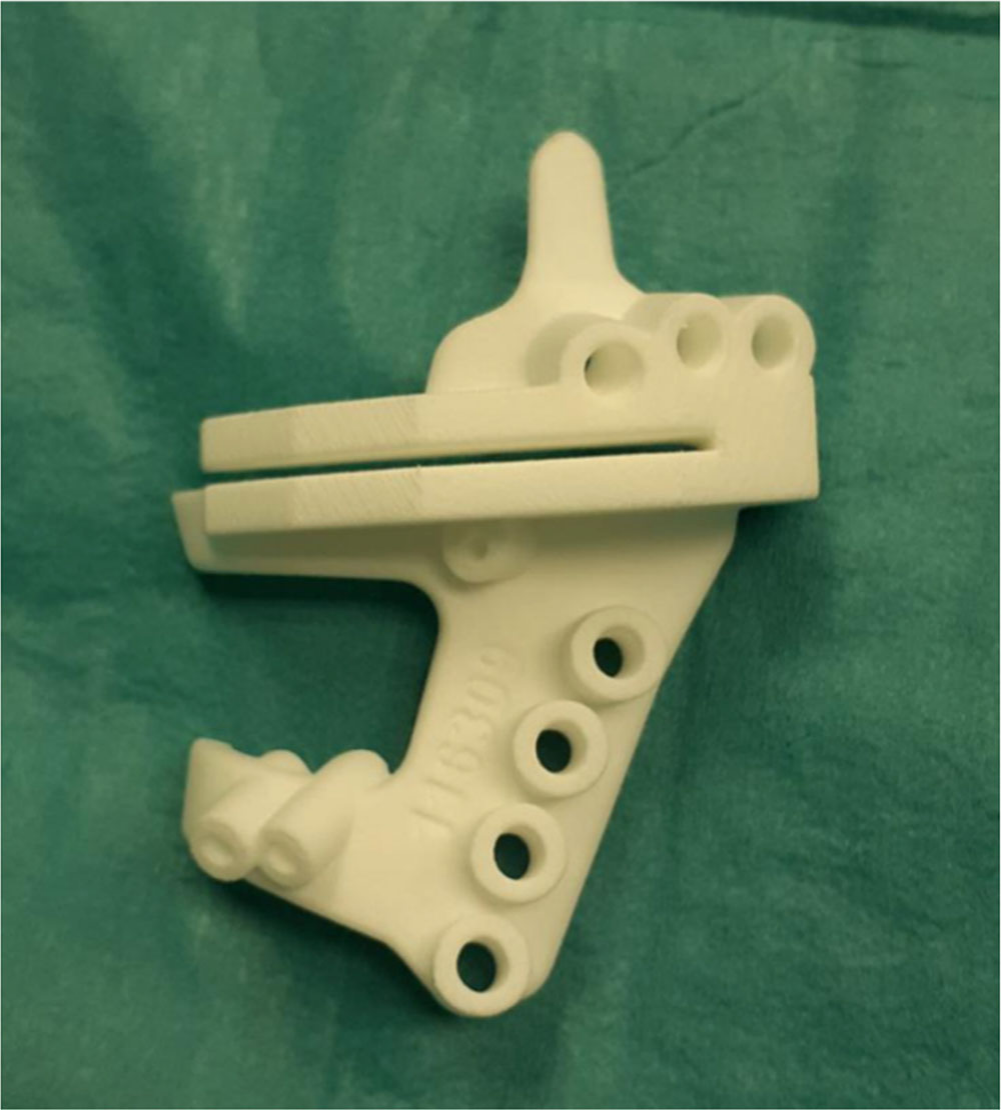
3D‐PSI‐printed guide PA2200. 3D‐PSI, 3D Guides Printed.

The 3D‐PSIs (medical device Class IIa) were manufactured via polyamide PA2200 with a FORMIGA P 110 Velocis (EOS, Germany) by selective laser sintering technology as the main material. Sterilization was conducted internally in our centre with ethylene oxide autoclaving (Figure 2).

### Statistical analysis

For normally distributed continuous variables, the mean was used as the central tendency measure, and the standard deviation (SD) was used as the measure of variance.

To assess the distribution of variables between different groups, the Shapiro‒Wilk test was used, and the results were considered statistically significant if the *p* value was less than 0.05.

A descriptive study of the variables was performed via SPSS v29.0 and RStudio 2023.04.21 with R version 4.3.0.

## RESULTS

A total of 30 patients were examined and divided into control and 3D guide groups.

The patients' mean age was 50.8 years (SD 8.67), and their body mass index (BMI) was 29.91 (SD 5.01). The 3D‐Guide group's mean age was 52.64 years (SD 6.45), and the mean BMI was 29.87 years (SD 4.25). The mean age of the control group (conventional technique) was 49.19 years (SD 10.17), and the mean BMI was 29.93 years (SD 5.74).

Differences between the grades of correction planned for MPTA and post‐operative MPTA between the two groups (MPTA dif). The values were 7.77° (SD 2.44) in the control group and 6.07° (SD 1.65) in the 3D‐Guide group, and for the post‐operative MPTA, the values were 89.54° (SD 1.06) and 89.14° (SD 0.83), respectively. The results, as presented in Table 3, revealed superior outcomes in the 3D‐Guide group. The study revealed a statistically significant difference between the intended correction and the final MPTA achieved after the surgical procedure. Specifically, the 3D‐Guide group presented more favourable results than the control group.

No differences were observed in the post‐operative HKA or in the grades of correction planned for HKA (*p* = 0.571) or the post‐operative HKA (*p* = 0.21) between the two groups (HKA dif.), as shown in Table 3.

The mean post‐operative JLCA was not significantly different (*p* = 0.546); in the control group, it was 0.97 (SD 0.95), and it was 0.75 (SD 0.56) in the 3D‐Guide group.

Additionally, the surgical time (measured in minutes) and radiation exposure (measured in seconds) were evaluated. Statistically significant results (*p* < 0.05) were observed with respect to the duration of radiation exposure, with 23.5 s in the control group and 8 s in the 3D‐Guide group. There was no difference in surgical time (Table 4). No complications were observed.

**Table 4:** A mean value (±standard deviation) is used to show the variables with normal distributions.

	Control (conventional technique), *N* = 15	3D guide, *N* = 15	*p*
JLCA preop	2.48 (2.13)	1.84 (1.47)	0.454
JLCA postop	0.97 (0.95)	0.75 (0.56)	0.546
MPTA preop	81.94 (1.62)	83.07 (1.59)	0.0875
MPTA postop	89.54° (1.06)	89.14° (0.83)	0.198^1^
MPTA dif	7.77° (2.44)	6.07° (1.65)	0.041^1^
HKA preop	170.52° (3.36)	172.21° (2.36)	0.126
HKA postop	178.7° (1.68)	178.4° (1.44)	0.5711
HKA dif	7.59° (2.46)	6.25° (2.92)	0.211
TIME IQ (min)	60.69 (8.89)	53.43 (11.69)	0.0691
TIME Rx (s)	23.5 (10.74)	8 (4.51)	0.0000341
KOOS pre	47.19 (14.16)	41.36 (16.19)	0.307
KOOS post 3 months	87.86 (5.64)	88.46 (3.53)	0.741
KOOS post 1 year	91.5 (5.72)	88.57 (8.81)	0.515

Abbreviations: HKA, hip–knee–angle; JLCA, joint line convergence angle; KOOS, Knee Injury and Osteoarthritis Outcome Score; MPTA, mechanical proximal tibial angle.

In terms of the clinical score (KOOS) evaluated at the preoperative stage and at 3 months and 1 year after surgery, no significant differences were found among the groups. The study did not identify any notable variations in the KOOS scores between the different groups under investigation (Table 4).

## DISCUSSION

The study successfully met its primary objective, revealing no difference between the techniques. These findings demonstrate that the accuracy of OWHTO via 3D guidance is comparable to that of the traditional method. The study demonstrated that the PSI system was able to achieve correction during the osteotomy procedure with a good level of accuracy [31] but was no better than the conventional technique. The results of the study are in line with the scientific debate on the topic.

In a single‐centre, retrospective, observational study, Predescu et al. [28] included 25 consecutive patients who underwent OWHTO via PSI and reported that the use of PSI in OWHTO allows accurate achievement of the desired correction. A recent meta‐analysis by Pang et al. [26] revealed results similar to those of our study. Their meta‐analyses aimed to determine whether PSI could improve the accuracy of correction in HTO. A systematic search was conducted via online databases, and a total of 466 patients were included in 11 papers that met the inclusion criteria. On the basis of the analysed data, they found no significant difference between the designed target values and the post‐operative correction values of the HKA and MPTA, concluding that while the PSI is accurate, it is not necessary for typical HTO.

Another study that demonstrated the high accuracy of 3D PSI was by Fayard et al. [12]. In this retrospective case‒control study, 49 patients with isolated medial OA of the knee who underwent OWHTO with a 3D guide were compared with 38 patients who underwent the procedure with the standard technique. The study revealed that the 3D guide technique was significantly more accurate in achieving the planned HKA in OWHTO.

The ESSKA consensus group recommends the use of preoperative planning in osteotomy surgery to determine the expected bone morphology after correction of the coronal alignment to ascertain the optimal technique and level of osteotomy for surgical correction [10].

This study supports these recommendations and confirms that 3D preoperative planning is crucial in defining the necessary correction.

To the best of our knowledge, few studies have compared 3D‐guided osteotomy with conventional techniques, such as that of Abdelhameed et al. [1], who reported that the free‐hand method is as precise as PSI in terms of reliability, achieving planned correction in knee corrective osteotomies. This finding is of paramount importance, as it highlights the effectiveness and trustworthiness of the 3D‐guide system in guiding and controlling OWHTO procedures.

It is crucial to gain experience with conventional techniques prior to adopting the use of PSI, which may be a demanding technique. The adoption of PSI for basic OWHTO is recommended for experienced surgeons who require familiarization prior to using the technique for more demanding cases.

Nuanced presentations with multiplanar deformities (especially including elements such as torsion and intra‐articular malunion) are ideal situations for PSIs in the hands of experienced surgeons, who have increased the learning curve in this technique [10].

Another finding of this study was the disparity observed between the planned correction grades of the MPTA and the MPTA post‐operatively, referred to as the difference in the MPTA. This discovery underscores the potential benefits of utilizing the PSI system in achieving improved accuracy and alignment in MPTA correction surgeries.

This approach could help reduce under‐ or overcorrections because it is the main reason for clinical failure in OWHTO, and the leading principle in such procedures should be to achieve the highest accuracy possible [17].

The study did not find significant differences in the post‐operative HKA or JLCA measurements between the two groups. The HKA is influenced by bone alignment and soft tissue status, as it incorporates the effect of JLCA values.

The values indicate comparable outcomes in terms of HKA alignment and the achieved correction grades between the two groups. This finding demonstrated that the use of the 3D guide did not lead to significant differences in HKA alignment compared with the control group.

The high accuracy of HKA correction via the PSI system presented here was confirmed [17].

Further investigation may be necessary to identify additional factors that could influence HKA alignment and the effectiveness of the 3D guide system.

These results are in line with those of previous studies. A systematic review by Aman et al. [4], with the purpose of evaluating the accuracy and precision of post‐operative coronal plane alignment via 3D‐printed PSI in the setting of proximal tibial or distal femoral osteotomies, revealed that patients who underwent distal femoral osteotomy or proximal tibial osteotomy procedures with 3D‐printed patient‐specific cutting guides presented highly accurate coronal plane alignment with a low rate of outliers.

Digitally planned and executed osteotomies under 3D‐printed osteotomy positioning guides help surgeons reduce surgical time by performing tibial opening wedge osteotomy [4, 27, 33].

In this study, in relation to the time of surgery, no statistically significant results (*p* < 0.05) were found.

However, the authors thought that the surgical time could be considered a matter of time and could have a clinical effect. There was a reduction of approximately 11.69 min in the surgical duration for the 3D‐Guide group. From our point of view, the shorter surgical timing associated with the PSI‐Guide system might suggest potential clinical benefits for the patient, such as decreased anaesthesia time, ischaemia time, and reduced operative risks and complications. Additionally, considering that all patients are in the outpatient setting, we cannot prove this in this study; therefore, more investigations are necessary.

Statistically significant results (*p* < 0.05) were observed in the study regarding the time of radiation exposure, specifically in the 3D‐Guide group. The findings revealed that the 3D‐Guide group experienced a significantly shorter radiation exposure time (8 s) than the control group. This suggests that during the surgical procedure, the utilization of the PSI‐Guide system led to a significant reduction in X‐ray exposure, following previous results in the literature [4, 27, 33], adding potential benefits to improve safety for both patients and healthcare professionals involved in the procedure [22, 28].

With respect to improvements in clinical outcomes, we can confirm that OWHTO is a useful and safe procedure [19, 20]. However, no significant differences were found among the groups regarding the clinical outcome (KOOS), which was evaluated at 3 months and 1 year after surgery. The study results indicate that the outcomes, as measured by the KOOS score, were comparable between the groups. This suggests that the use of the PSI‐Guide did not have a significant effect on the post‐operative clinical scores compared with those of the control group. Importantly, the number of patients evaluated was small; therefore, more patients are needed for further assessment. In addition, further analysis and studies with long‐term follow‐up may be needed to assess the impact of the PSI‐Guide on clinical scores beyond the 1‐year timeframe fully.

This study has several limitations. First, the sample size was limited, as six patients were lost during the study, and data from only 30 patients were collected between November 2019 and February 2023 because of the impact of the COVID‐19 pandemic. A larger sample size is needed to detect small differences more accurately. Additionally, outcomes in the sagittal plane, such as the tibial slope, were not analysed.

Another limitation was the timing of the clinical follow‐up. Clinical outcomes were only evaluated over a minimum 1‐year period, so a longer follow‐up is needed to obtain more accurate results. However, the primary aim of this investigation was to identify any immediate radiological issues associated with this relatively new technique.

Finally, the comparison was based on standard X‐rays, which provide only 2D analysis and are subject to errors related to limb rotation and distance. Despite this, full‐limb weight‐bearing views remain the gold standard with an acceptable margin of error [9, 34].

## CONCLUSIONS

According to the results of this study, 3D‐printed guided OWHTO achieves good correction, with results compared with those of the conventional free‐handed technique. A shorter radiation exposure time and inferior surgical time are observed in 3D‐printed guided OWHTO. Further research is needed to explore the clinical implications and long‐term effects of these differences, the economic benefits of this technique, and further correlations with bone union.

## AUTHOR CONTRIBUTIONS

Giovanni Grillo: Conceived the analysis, collected the data and wrote the paper. Alexandre Coelho: Collected the data and wrote the paper. Xavier Pelfort: Collected the data. Ferran Fillat‐Gomà: Contributed with analysis tools. Arnau Verdaguer Figuerola: Collected the data. Sònia Carbó Cerdán: Collected the data. Juan Manuel Peñalver: Collected the data. Christian Yela‐Verdú: Conceived the analysis and wrote the paper.

## CONFLICT OF INTEREST STATEMENT

The authors declare no conflicts of interest.

## ETHICS STATEMENT

The study received institutional review board approval from the ethics committee (OTV‐3D/CIR2019007). Informed consent was obtained from all individual participants.
